# Assessment of hypertension management and control: a registry-based observational study in two municipalities in Cuba

**DOI:** 10.1186/s12872-019-1006-6

**Published:** 2019-01-30

**Authors:** Esteban Londoño Agudelo, Armando Rodríguez Salvá, Addys Díaz Piñera, René García Roche, Pol De Vos, Tullia Battaglioli, Patrick Van der Stuyft

**Affiliations:** 1 Department of Public Health, Institute of Tropical Medicine, St. Rochusstraat 43, 2000 Antwerp, Belgium; 2 Centro de Epidemiología y Salud Ambiental, Instituto Nacional de Higiene, Epidemiología y Microbiología (INHEM), Infanta No. 1158 e/ Llinas y Clavel, Centro Habana, 10300 La Habana, Cuba; 3 Institute for Global Health and Development, Queen Margaret University, Musselburgh - Edinburgh, EH21 6UU UK; 4 Faculty of Medicine and Health Sciences. Department of Public Health and Primary Care, Ghent University, C. Heymanslaan 10, ingang 42, verdieping 5, 9000 Ghent, Belgium

**Keywords:** Hypertension, Cuba, Chronic disease, Treatment, Antihypertensive agents, Primary health care

## Abstract

**Background:**

To determine the prevalence of hypertension treatment and control among hypertensive patients in the Cuban municipalities of Cardenas and Santiago and to explore the main associated predictors.

**Methods:**

Cross-sectional study, with multistage cluster sampling, conducted between February 2012 and January 2013 in two Cuban municipalities. We interviewed and measured blood pressure in 1333 hypertensive patients aged 18 years or older. Hypertension control was defined as blood pressure lower than 140/90 mmHg.

**Results:**

The mean age ± standard deviation (SD) of participants was 59.8 ± 14 years, the mean systolic and diastolic blood pressure ± SD was 130.0 ± 14.4 and 83.1 ± 9.0 mmHg respectively. The majority of patients (91, 95%CI 90–93) were on pharmacological treatment, 49% with a combination of 2 or more classes of drugs. Among diagnosed hypertensive patients 58% (95%CI 55–61) had controlled hypertension. There was no association between hypertension control and gender, age and socio-economic condition. Levels of hypertension control depended on health area and control furthermore was positively associated with post-primary education, not being obese and white ethnicity: adjusted Odds Ratio (95% CI) 1.71 (1.26–2.34), 1.43 (1.09–1.88) and 1.41 (1.09–1.81) respectively.

**Conclusions:**

The observed figures are outstanding at the international level and illustrate that hypertension treatment and control are achievable in a resource-constrained setting such as Cuba. The country’s primary health care approach and social equity in access to health care can be seen as key determinants of this success. Nevertheless, there is still room for improvement, as over a third of patients did not have controlled hypertension.

## Background

Non-communicable diseases (NCDs) account for 63% of global mortality nowadays and it is predicted that they will account for 69% of global deaths by 2030 [1]. They are significantly related to preventable early mortality and disability and they are especially worrying for developing countries, where their incidence is increasing disproportionately. According to the Pan American Health Organization, non-communicable diseases are the first cause of death and disability in the Americas region, generating almost 4 million deaths annually [2].

Cardiovascular diseases (CVD) are the leading cause of death around the world and uncontrolled hypertension is a related cause of 7.6 million deaths each year [3]. Furthermore, approximately 51% of deaths due to stroke and 45% of deaths due to ischaemic heart disease are attributable to systolic hypertension. The risk of dying from hypertension at all ages is more than double in low and middle income countries (LMIC) compared to high income countries (HIC) [4].

Notwithstanding, the majority of health systems provide inadequate chronic diseases management, with health services mainly designed to provide acute curative care. This global trend has been termed “the tyranny of the urgent” [5]. Chronic care, especially in developing countries, is often reduced to the belated management of acute exacerbations of chronic illnesses in specialized settings and at high costs. Not surprisingly, most out-of-pocket health payments and catastrophic expenditure are related to chronic conditions, in particular especially to costly but avoidable complications such as kidney failure, heart attack and stroke [6–8].

Cuba went through an epidemiological transition from the mid-twentieth century onwards. Today, chronic diseases account for 86% of total deaths in the country and CVD are responsible for 39% of the overall mortality [9]. In 2013, the death rate from CVD was 202.9 per 100.000 population [10], while in the latest nationwide survey hypertension prevalence was estimated at 30.9% of the population above 15 years [11]. The ongoing process of urbanization and lifestyle changes contributes to a further increasing burden of CVD [12].

Cuba is internationally recognized for having a well-organized national health system based on a primary health care (PHC) approach and it can be considered an excellent setting to study the potential of low-resource health systems to develop comprehensive programs for chronic care. The Cuban health system not only has achieved excellent control of communicable diseases but also presented one of the best hypertension control figures in the world [13]. Nevertheless, there remain problems with regard to quality and long-term continuity of care provided for chronic patients. The last internationally published study on hypertension control in the country, carried out in the Cienfuegos province in 2001–2002, reported a control rate of 62% among treated hypertensive patients [13]. Since then, Cuba experienced socioeconomic and political changes, possibly affecting population’s health. More than a decade after the latest report, an update is needed.

The present study primarily aims at determining the current proportion of treated and controlled hypertensive patients in two different geographical areas of the country (Cardenas and Santiago municipalities). Moreover, predictors associated with achieving hypertension control were explored.

## Methods

This cross-sectional study was conducted between February 2012 and January 2013 in the Cuban municipalities of Cardenas and Santiago (total population in 2012: 142.369 and 513.784 respectively) [14]. Health areas in the Cuban PHC system consist of family doctor/nurse practices (FDNP) and policlinics. FDNP take responsibility for population health and are in charge of carrying out individual and community risk assessment [15]. They are the first entry point to the health care system. Policlinics provide diagnostic and support services to FDNP and specialized care. A health area is comprised of one policlinic and around 30 FDNP and counts approximately 30.000 inhabitants. The entire population is registered with a FDNP.

The study municipalities and the health areas within each municipality were purposively selected on the basis of feasibility and the commitment of local health authorities to carry out the study and to implement subsequently interventions for quality improvement of the hypertension management program. In each municipality, two urban health areas were selected: “Julio Antonio Echeverria (JAE)” and “Moncada” in Cardenas and “Grimau” and “Finlay” in Santiago. In each health area, 14 FDNP were randomly selected. In each of these FDNP, 25 hypertensive patients were selected by simple random sampling from the family doctor register. Patients were included if they were aged 18 years or older, had a confirmed diagnosis of hypertension documented in their medical records and provided their written informed consent. Thus, a total of 1400 hypertensive patients were sampled (700 per municipality, 350 per health area), allowing to estimate the proportion of patients with controlled hypertension (anticipated to be at least 50%) with a confidence level of 95%, a precision of 5% and a cluster effect of 2.5.

Data collection was done by a group of junior researchers of the Cuban National Institute of Hygiene and Epidemiology (INHEM). In order to guarantee reliability and consistency, they received a 3 days training at INHEM and had to pass a standardized test on blood pressure measurement. Door to door visits in the homes of the selected patients, during which blood pressure measurement and interviews were carried out, were made by research staff. In case the person was not found at home, two further visits were made before another patient was selected. Blood pressure was measured three times, from the right arm in a sitting posture, using a mercury manometer, following international standardized recommendations for blood pressure measurement in population surveys [16, 17]. The mean of the last two readings was calculated and hypertension was defined as controlled if the mean systolic and diastolic blood pressure were respectively lower than 140 and 90 mmHg. All participants were interviewed on their previous and current health problems, anti-hypertensive pharmacological treatment and health seeking behaviour using a structured questionnaire. The main variables measured during the survey were assessed by self-report: ethnicity (white / non-white, comprising mestizo and black); post-primary education (yes: university, technical college or secondary school / no: did not finish secondary school); paid job (yes: state worker, self-employed / no: housewife, student, unemployed and retired); medical history of diabetes (yes / no); medical history of heart disease (yes / no). To determine levels of adherence to pharmacological treatment, we applied the four-item Medication Adherence Questionnaire (MAQ) by Morisky et al. [18].

Participants’ weight and height were also measured. Body weight was measured to ±0.1 kg using electronic scales, standing height was measured to ± 0.01 m using a wall mounted stadiometer and body-mass index (BMI) was calculated as weight/height2 (kg/m2). Based on Word Health Organization criteria [19, 20] nutritional status was classified on the basis of BMI: underweight: below 18.5; normal weight: 18.5–24.9; overweight: 25–29.9; obesity: ≥30. BMI was dichotomised as obese/non-obese for further analysis.

Data were double-entered in a Microsoft Access 2000 database and analysed using the Statistical Package for Social Sciences (SPSS) V.23 (SPSS Inc., Chicago, IL, USA). Means and standard deviations and proportions with 95% confidence intervals (95%CI) were calculated for continuous and categorical variables respectively. The association between hypertension control and socio-demographic and clinical factors was explored using the Chi-squared and the t test for categorical and continuous variables respectively. *P* values less than 0.05 were considered significant. Potential predictors for controlled hypertension were included in a multivariate logistic regression model if they were statically significant in bivariate analysis (conservatively taking *p* < 0.1 as threshold) or a priori considered of relevance. Unadjusted and adjusted Odds Ratios (ORs) and their 95%CI were calculated, taking into account the clustered/multistage study design.

## Results

**Fig. 1:**
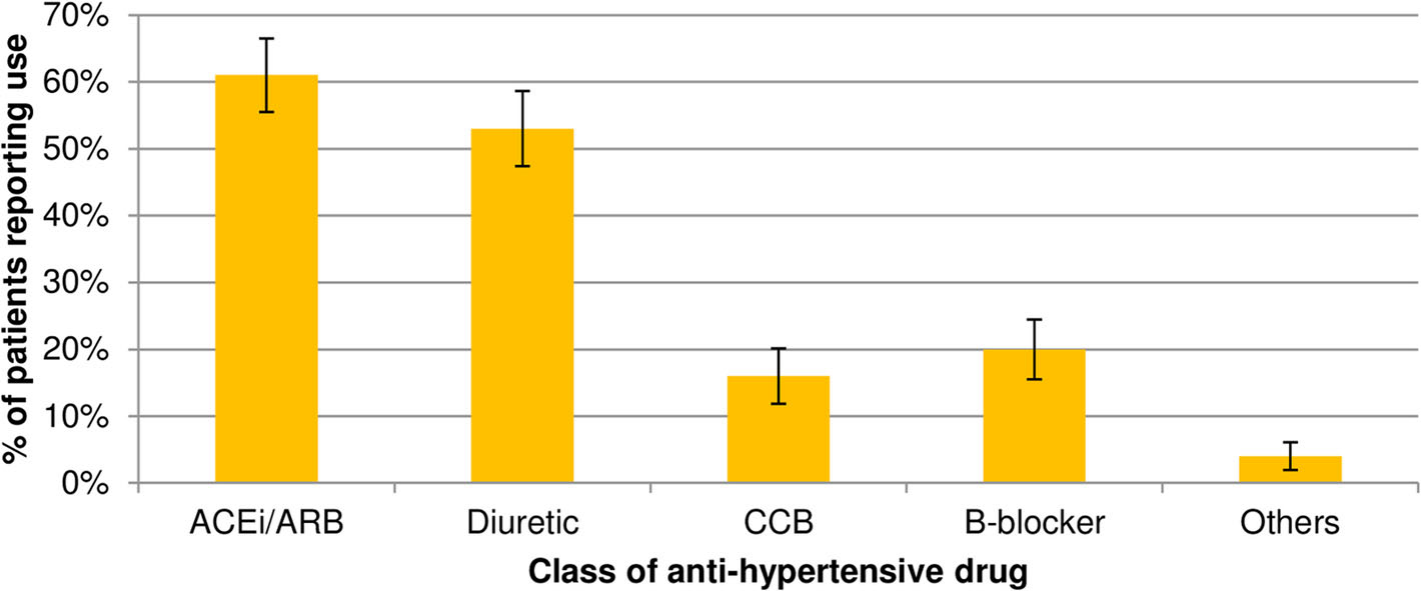
Prevalence of use of main classes of anti-hypertensive drugs. Cardenas and Santiago municipalities, Cuba, 2012–2013. Error bars represent the 95% confidence intervals. ACEi/ARB = Angiotensin Converting Enzyme inhibitor / Angiotensin II Receptor Blocker; B-Blocker = Beta-blocker.; CCB = Calcium Channel Blocker

**Fig. 2:**
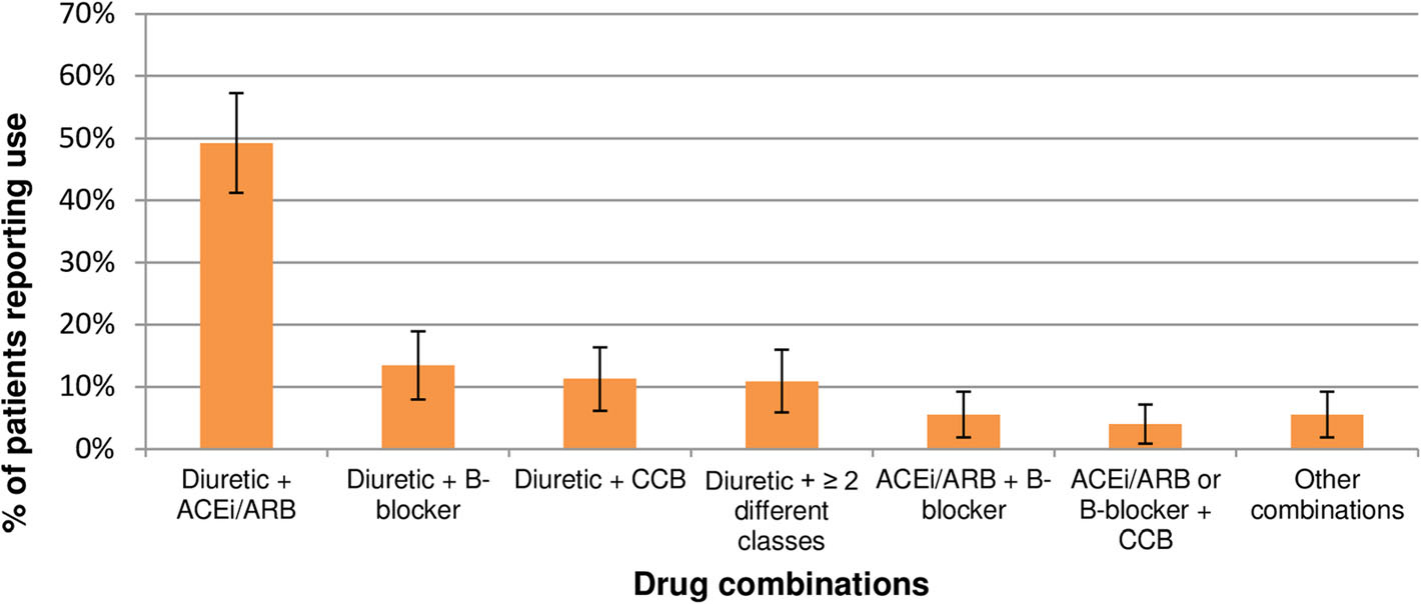
Prevalence of use of different anti-hypertensive drug combinations. Cardenas and Santiago municipalities, Cuba, 2012–2013. Error bars represent the 95% confidence intervals. ACEi/ARB = Angiotensin Converting Enzyme inhibitor / Angiotensin II Receptor Blocker; B-Blocker = Beta-blocker.; CCB = Calcium Channel Blocker

Sixty-seven sampled patients (5%), mostly from JAE health area, were not found at home after 3 visits but were not replaced due to operational constraints. Hence, the total number of respondents was 1333 (Table 1). There was a higher percentage of women, especially in the health areas of Santiago. The mean age of participants was 59.8 years and females were older than males (61.2 vs 58.0 years, *p* < 0.001). Based on self-report, 43% of the sample was white, 29% mestizo and 28% black. High levels of health care utilization were observed: 42% of patients had a contact during the 3 months before the survey; 71% (95% CI, 68–74%) of these contacts were with first-line health services. The mean blood pressure in the study population was 130.0/83.0 mmHg. Controlled hypertension was found in 773 patients (58, 95%CI 55–61) (Table 1). The majority of respondents (91%) reported receiving anti-hypertensive pharmacological treatment. Among those taking medication 49% (95% CI, 46–52) were fully complying with the treatment. The mean BMI ± SD was 26.5 ± 4.6. The most frequently used classes of drugs were angiotensin-converting enzyme inhibitors/angiotensin II receptor blockers (ACEi/ARB), followed by diuretics, beta-blockers and calcium channel blockers (CCB) (Fig. 1). Overall, 49% (95%CI 46–52) of patients receiving treatment were taking a combination of two or more classes of drugs. Diuretics plus ACE/ARB was the most frequently used combination (49, 95%CI 45–53), followed by diuretics plus beta blockers (13, 95%CI 11–16) and diuretics plus CCB (11, 95%CI 9–14) (Fig. 2).

**Table 1:** General characteristics, blood pressure parameters, hypertension treatment and control, by health area, in hypertensive patients in the municipalities of Cardenas and Santiago, Cuba, 2012–2013

	Total (*n* = 1333)		Cardenas				Santiago			
	No. (%) / mean	95% CI	JAE (No. = 292)		Moncada (No. = 346)		Finlay (No. = 351)		Grimau (No. = 344)	
			No. (%)/ mean	95% CI	No. (%) / mean	95% CI	No. (%) / mean	95% CI	No. (%) / mean	95% CI
Female sex	819 (61)	59–64	164 (56)	50–62	194 (56)	51–61	218 (62)	57–67	243 (71)	66–75
Age (years)	59.8	59.1–60.6	58.7	57.1–60.3	59.2	57.9–60.7	62.5	61.0–63.9	58.7	57.2–60.2
Non-white ethnicity	758 (57)	54–60	92 (32)	26–37	127 (37)	7–14	236 (67)	62–72	303 (88)	85–92
Post-primary education	1128 (85)	83–87	265 (91)	87–94	261 (75)	71–80	310 (88)	85–92	292 (85)	81–89
Single civil status	571 (43)	40–45	112 (38)	33–44	130 (38)	32–43	174 (50)	44–55	155 (45)	40–50
Paid job	522 (39)	37–42	143 (49)	43–55	127 (37)	32–42	125 (36)	31–41	127 (37)	32–42
Body Mass Index										
Underweight	27 (2)	1–3*	5 (2)	1–4	10 (3)*	1–5*	6 (2)	1–4*	6 (2)	1–4*
Normal weight	518 (39)	36–41	113 (39)	33–44	113 (33)	28–38	144 (41)	36–46	148 (43)	38–48
Overweight	523 (39)	37–42	125 (43)	37–48	139 (40)	35–45	133 (38)	33–43	126 (37)	32–42
Obese	265 (20)	18–22	49 (17)	12–21	84 (24)	20–29	68 (19)	15–24	64 (19)	14–23
Diabetes	207 (16)	14–17	33 (11)	8–15	40 (12)	8–15	69 (20)	16–24	65 (19)	15–23
Coronary heart disease	185 (14)	12–16	26 (9)	6–12	54 (16)	12–19	65 (19)	14–23	40 (12)	8–15
Blood pressure (mmHg)										
Systolic	130.0	129.3–130.8	128.9	127.3–130.5	131.7	128.7–131.7	129.2	127.8–130.6	130.2	128.7–131.7
Diastolic	83.1	82.6–83.6	81.5	80.4–82.5	83.8	82.3–84.8	84.2	83.3–85.2	82.6	81.7–83.5
On anti-hypertensive pharmacological treatment	1218 (91)	90–93	284 (97)	95–99	318 (92)	89–95	318 (91)	87–93	298 (87)	83–92
Controlled hypertension	773 (58)	55–61	191 (65)	60–71	186 (54)	48–59	187 (53)	48–59	209 (61)	55–66

*CI* confidence interval, *JAE* José Antonio Echeverría. * Exact confidence interval

Table 2. The proportion of patients with hypertension control was significantly higher in individuals with post-primary education, that were non-obese and had white ethnicity. Given the results of bivariate analysis, which showed that the percentage of patients with controlled hypertension among underweight, normal weight and overweight individuals was similar: respectively 59% (16/27), 60% (308/518) and 60% (315/523) against 51% (134/265) in obese individuals, BMI was dichotomised as obese/non-obese. Municipality was not related to hypertension control (59% in Cárdenas, 57% in Santiago, *p* = 0.44) but levels of hypertension control depended on health area. Gender, age, civil status, labour activity, receiving anti-hypertensive drugs, compliance with pharmacological treatment and personal history of coronary heart disease or diabetes were not significantly associated with hypertension control. Among the 115 not receiving treatment 55% (95% CI, 46–64) had controlled hypertension. In multivariate analysis, the variables which exerted significant influence on hypertension control were: post-primary education, obesity and white ethnicity (Table 2).

**Table 2:** Predictors of controlled hypertension among hypertensive patients in the municipalities of Cardenas and Santiago, Cuba, 2012–2013

Predictor	Controlled hypertension, No. (%)		Unadjusted OR (95% CI)	Adjusted OR (95%CI)
	No	Yes		
Sex				
Male	224 (44)	290 (56)	Reference	–
Female	336 (41)	483 (59)	1.11 (0.89–1.39)	
Age in years				
< 65	332 (41)	475 (59)	1.10 (0.88–1.37)	–
≥ 65	228 (43)	298 (58)	Reference	
Ethnicity				
Non-white	338 (45)	420 (55)	Reference	Ref.
White	222 (39)	353 (61)	1.28 (1.03–1.59)	1.39 (1.08–1.79)
Post-primary Education				
No	109 (53)	96 (47)	Reference	Ref.
Yes	451 (40)	677 (60)	1.70 (1.26–2.30)	1.72 (1.26–2.35)
Civil status				
Single	241 (42)	330 (58)	Reference	–
Married/Partner	319 (42)	443 (58)	1.01 (0.81–1.26)	
Paid job				
No	222 (43)	300 (58)	Reference	–
Yes	338 (42)	473 (58)	0.97 (0.77–1.20)	
Obesity				
Yes	131 (49)	134 (51)	Reference	Ref.
No	429 (40)	639 (60)	1.46 (1.11–1.91)	1.43 (1.09–1.89)
Diabetes				
Yes	89 (43)	118 (57)	Reference	–
No	471 (42)	655 (58)	1.05 (0.78–1.42)	
Coronary heart disease				
Yes	79 (43)	106 (57)	Reference	–
No	481 (42)	667 (58)	1.03 (0.75–1.42)	
On anti-hypertensive pharmacological treatment				
===No	52 (45)	63 (55)	Reference	Ref.
Yes, non-adherent	281 (45)	341 (55)	1.00 (0.7–1.5)	1.06 (0.70–1.59)
Yes, adherent	227 (38)	369 (62)	1.34 (0.90–2.0)	1.35 (0.90–2.05)
Health area				
JAE	101 (35)	191 (65)	Reference	Ref.
Finlay	164 (47)	187 (53)	0.60 (0.45–0.81)	0.75 (0.54–1.04)
Grimau	135 (39)	209 (61)	0.82 (0.60–1.11)	1.11 (0.78–1.56)
Moncada	160 (46)	186 (54)	0.62 (0.46–0.83)	0.71 (0.52–0.98)

*CI* confidence interval, *JAE* José Antonio Echeverría

## Discussion

This study found a high prevalence of pharmacological treatment and blood pressure control among diagnosed hypertensive patients in two Cuban municipalities. Almost half of patients receiving treatment were taking two or more antihypertensive drugs. Hypertension control varied by health area within a municipality. Having post-primary education, not being obese and being of white ethnicity were positively associated with hypertension control.

Given that in Cuba the entire population is registered with a FDNP, who must carry out an individual risk assessment of the recorded population on a yearly basis, registers of patients provided by the Ministry of Health reflect the actual population’s health status. This is corroborated by the findings of the Third National Survey on Risk Factors in Cuba 2010–2011 where the prevalence of diagnosed hypertensive patients in the population above 15 years (2010) was 22.4% vs 20.4% found in the Ministry of Health registers [11]. The study population consisted of a sample drawn from two of the 168 Cuban municipalities and thus it may not represent the national situation, in particular with respect to rural areas. Nevertheless, the found prevalence figures are consistent with national surveys [11] and an earlier similar study in the Cuban province of Cienfuegos [13]. In order to minimize errors, international standardized recommendations for blood pressure measurement in population surveys were followed [16, 17]. However, blood pressure control was measured in a single visit, with the possibility of some false-positive uncontrolled hypertensive patients, resulting in an underestimation of hypertension control. Except for obesity, presence of comorbidities was self-reported without confirmation on clinical records or with diagnostic tests, which can be a limitation for their analysis as predictors. The use of self-report scales for measuring medication adherence also has potential limitations, especially regarding patients’ ability to understand the items and willingness to disclose information, which can affect questionnaire validity [21]. Moreover, the 4-item Morisky MAQ [18] is only able to address barriers to medication-taking but not self-efficacy [21, 22]. Nevertheless, this test, validated in the USA with hypertensive patients, presented a reasonable specificity in identifying non-adherent behaviour [23]. Another limitation is that factors such as smoking status, duration of hypertension or lipid profile, which besides being etiologically related to hypertension could also be associated with its control, were not included in the study.

The high prevalence of hypertension treatment and control among diagnosed patients found in this study is consistent with a previous study carried out in the Cuban province of Cienfuegos in 2001–2002 [13], suggesting more than a decade of sustainable outcomes. The recent PURE study [3] found an average prevalence of hypertension control among diagnosed patients receiving treatment of 41% in HIC and 27% in LMIC. Hence, the prevalence of hypertension control in Cuba found in the present study (58%) is considerably higher than the average in HIC and double that of LMIC. The figure is similar to what found in the Health Survey for England 2015, where 62% of treated patients had their blood pressure under control [24]. A study comparing hypertension management in 20 countries among both aware and unaware systolic hypertensive patients, reports that the United States had the best age-standardized prevalence of systolic hypertension treatment and control in patients aged 35 to 84 years (81% for treatment and 59% for control) followed by Jordan (71 and 38%) and England (54 and 32%) [25]. Due to the heterogeneity of methods among different studies, conclusions based on such comparisons should be interpreted with caution. Notwithstanding internationally outstanding figures, still more than a third of hypertensive patients did not have controlled hypertension, which warrants the attention of the Cuban health authorities.

Hypertension control at population level has been forwarded as a correlate measure of how well a health system functions, given that this condition is entirely dependent on the health system for its care and control [26]. Constrains to provide integrated and quality chronic care have been associated with weak national health systems and fragmented health-care services [1, 27, 28]. For instance, in Sub-Saharan Africa, a region characterized by weak health systems, hypertension remains largely under-diagnosed and under-treated [26]. From a health system perspective, at least three key drivers of successful hypertension control in Cuba can be identified. First and foremost, a PHC approach: Cuba, despite being a resource-constrained setting, ensures free and accessible quality care through FDNP [15] and is recognized for having a well-organized national health system that ensures inter-sectorial actions to promote health and wellbeing [12, 13, 29]. As underlined by the European Forum for Primary Care, a strong PHC system is better prepared to provide comprehensive health care and effectively co-ordinate the follow-up of chronic conditions [30]. Besides Cuba, two other Latin-American countries, Brazil and Costa Rica, provide evidence of the effectiveness, efficiency and relevance of the PHC approach for the management of chronic conditions [28, 31–33]. Second, the high percentage of patients receiving pharmacological treatment found in this study confirms the reported availability, proper procurement and distribution of essential anti-hypertensive drugs in Cuba [29]. Third, Cuba has one of the highest densities of physicians in the world: 67.2 per 10,000 population, only surpassed by rich countries such as Monaco and Qatar [34]. Moreover, family doctors in charge of PHC services are specialists, with at least 3 years of postgraduate training [15]. In summary, the main health system barriers to achieve blood-pressure control pinpointed by Ibrahim and Damasceno (namely scarce human resources, absence of a national policy for the control of NCDs, poor training of health personnel, overburdened and disorganized PHC and a deficient procurement and distribution of essential antihypertensive drugs) [35], have been overcome in the Cuban health system. Notwithstanding, the association of hypertension control with living in a specific health area suggests that hypertension control could be impacted at community level by improving health services functioning at micro-level.

Appropriate drug titration and combination therapies are also key elements for achieving hypertension control. According to international guidelines, more than two-thirds of hypertensive patients require treatment with more than one antihypertensive drug to achieve blood pressure control [3, 36, 37]. The proportion of patients taking two or more types of antihypertensive drugs found in this study (49%) was high compared to average international figs. [3], suggesting that clinical inertia is not an important barrier affecting adequate antihypertensive therapy in Cuba. Overall, the combinations of antihypertensive drugs found in this study are aligned with international guidelines [36, 37]. Moreover, the type and frequency of medications used are more similar to the pattern reported in HIC than in LMIC [3]. Nevertheless, recent evidence showed that B-blockers are inferior to other drugs for the prevention of major CVD, stroke and renal failure [38] and they were not recommended by the Eight Joint National Committee for the initial treatment of hypertension [37]. Therefore, Cuban health authorities should take appropriate measures to address the high use of B-blockers and decrease their prescription, especially as monotherapy or initial treatment.

Being on pharmacological treatment was not significantly associated with hypertension control, but the vast majority of patients were on pharmacological treatment and control in other hypertensive may have been achieved by lifestyle modifications, which were not explored in depth in the present study. International literature widely describes the positive effects that sustained lifestyle modifications such as weight loss, diets rich in fruits/vegetables, and sodium (Na+) reduction have on blood pressure (BP) control [39]. Other non-pharmacological strategies recommended for patients with hypertension include tobacco cessation, decreased alcohol consumption and self-measured BP monitoring [40]. However, this study, did not explore the effect of non-pharmacological approaches to blood pressure control. Pharmacological treatment and adherence to it was included in the final multivariate model, but the difference between the individual categories was not significant. This could possibly be explained by inherent limitations of the Morisky test for capturing actual adherence, which have been signaled before.

The lack of association of hypertension control with age, gender and socio-economic conditions could be explained by the equity in access to health care services in Cuba irrespective of gender or social condition, where all adults receive at least one home visit of their family doctor/nurse per year, a frequency that increases according to specific risk profiles or disabilities [12]. The association between post-primary education and better hypertension control is consistent with other national and international reports [11, 41–44]. Furthermore, the high prevalence of post-primary education found in the study population (85%), is a significant factor related to achieving hypertension control in Cuba, as a higher education level is relevant for disease awareness and compliance to treatment [45, 46].

Obesity was found to be associated with poorer hypertension control. This is compatible with available evidence reporting a stable linear relation between adiposity and blood pressure, independent of age and body fat distribution [35]. High BMI alone is a very well established risk factor for hypertension and obese individuals have increased relative risk for CVD [36]. Obesity is a serious concern indeed for Cuba: in 2011, 45% of the Cuban population was overweight and 15% was obese [11].

In this study non-white hypertensive patients (mestizo or black) were more likely to have uncontrolled hypertension, regardless of socio-economic condition. Differences associated with ethnicity could be related to lifestyle differences or to genetic factors such salt sensitivity, which is more common in black people [35]. There are no specific studies on hypertension and cardiovascular complications among black communities in the Latin American and Caribbean region [47]. Studies done in United States showed that hypertension is more common, severe and leads to more clinical sequelae in African Americans compared to non-Hispanic whites [36]. Nevertheless, since African Americans also have a greater prevalence of other cardiovascular risk factors, hypertension has been mainly attributed to environmental and lifestyle factors and socio-economic condition rather that to genetically defined racial differences [35, 36].

Lowering blood pressure significantly reduces the risk of major cardiovascular events and all-cause mortality [38] and hypertension control at population level should be of the highest priority in all countries. Evidence also indicates that only well-functioning health systems are able to address NCDs effectively and equitably [48]. Based on the results reported here, a set of interventions - at health service and community level - aimed at increasing the effectiveness of hypertension management programs in both municipalities will be designed, implemented and evaluated.

## Conclusions

While there is still room for improvement, the figures observed in this study are outstanding at the international level and illustrate that hypertension treatment and control is achievable in a resource-constrained setting such as Cuba. The country’s primary health care approach and social equity in access to care can be seen as key to overcome system barriers to blood-pressure control and sustained clinical outcomes. This may inspire policy makers in other developing countries to adapt their public health systems’ set up and functioning to respond to the growing need for better chronic care.

## Abbreviations

ACEi: Angiotensin-converting enzyme inhibitors
ARB: Angiotensin II receptor blockers
B-Blocker: Beta-blocker
BMI: Body-mass index
CCB: Calcium channel blockers
CVD: Cardiovascular diseases
FDNP: Family doctor/nurse practices
HIC: High income countries
JAE: “Julio Antonio Echeverria” health area
LMIC: Low and middle income countries
MAQ: Medication Adherence Questionnaire
NCDs: Non-communicable diseases
PHC: Primary health care

## References

[1] Samb B, Desai N, Nishtar S, Mendis S, Bekedam H, Wright A (2010). Prevention and management of chronic disease: a litmus test for health-systems strengthening in low-income and middle-income countries. Lancet.

[2] Pan American Health Organization. Innovative Care for Chronic Conditions: organizing and delivering high quality Care for Chronic Noncommunicable Diseases in the Americas. Washington, DC: PAHO; 2013 https://www.paho.org/hq/dmdocuments/2013/PAHO-Innovate-Care-2013-Eng.pdf. Accessed 26 Jan 2019.

[3] Chow CK, Teo KK, Rangarajan S, Islam S, Gupta R, Avezum A (2013). Prevalence, awareness, treatment, and control of hypertension in rural and urban communities in high-, middle-, and low-income countries. JAMA.

[4] World Health Organization. Global health risks: mortality and burden of disease attributable to selected major risks. Geneva, Switzerland: WHO Press; 2009. http://www.who.int/healthinfo/global_burden_disease/GlobalHealthRisks_report_full.pdf. Accessed 26 Jan 2019.

[5] Bodenheimer T, Wagner EH, Grumbach K (2002). Improving primary care for patients with chronic illness. JAMA.

[6] Meraya AM, Raval AD, Sambamoorthi U (2015). Chronic condition combinations and health care expenditures and out-of-pocket spending burden among adults, medical expenditure panel survey, 2009 and 2011. Prev Chronic Dis.

[7] Islam MM, Yen L, Valderas JM, McRae IS (2014). Out-of-pocket expenditure by Australian seniors with chronic disease: the effect of specific diseases and morbidity clusters. BMC Public Health.

[8] Jaspers L, Colpani V, Chaker L, Van der Lee SJ, Muka T, Imo D (2015). The global impact of non-communicable diseases on households and impoverishment: a systematic review. Eur J Epidemiol.

[9] World Health Organization. Noncommunicable diseases country profiles 2014. Geneva, Switzerland: WHO document production services; 2014. http://apps.who.int/iris/bitstream/handle/10665/128038/9789241507509_eng.pdf;jsessionid=B8E69B0214D8E65022A4F1AE6117134D?sequence=1. Accessed 26 Jan 2019.

[10] Ministerio de Salud Pública. Anuario Estadístico de Salud 2013. La Habana, Cuba: Dirección de Registros Médicos y Estadísticas de Salud; 2014. http://files.sld.cu/dne/files/2014/05/anuario-2013-esp-e.pdf. Accessed 26 Jan 2019.

[11] Bonet-Gorbea M, Varona-Pérez P, ed. III Encuesta Nacional de Factores de Riesgo y Actividades Preventivas de Enfermedades No Transmisibles. Cuba 2010–2011. La Habana: Editorial Ciencias Médicas; 2014.

[12] Organización Panamericana de la Salud. Salud en las Américas, Edición de 2012: Volumen de países (Cuba). Washington, DC: PAHO; 2012. https://www.paho.org/salud-en-las-americas-2012/index.php?option=com_docman&view=download&category_slug=sa-2012-capitulos-pais-23&alias=199-cuba-199&Itemid=231&lang=en.

[13] Ordunez-Garcia P, Munoz JL, Pedraza D, Espinosa-Brito A, Silva LC, Cooper RS (2006). Success in control of hypertension in a low-resource setting: the Cuban experience. J Hypertens.

[14] Ministerio de Salud Pública. Anuario Estadístico de Salud 2012. La Habana, Cuba: Dirección de Registros Médicos y Estadísticas de Salud; 2013. http://files.sld.cu/dne/files/2013/04/anuario_2012.pdf. Accessed 26 Jan 2019.

[15] Ministerio de Salud Pública. Programa del médico y la enfermera de la familia. La Habana, Cuba: Editorial de Ciencias Médicas; 2011. http://files.sld.cu/sida/files/2012/01/programa-medico-y-enfermera-2011-vigente.pdf. Accessed 26 Jan 2019.

[16] Russell V. Luepker, Alun Evans, Paul McKeigue, K.Srinath Reddy. Cardiovascular survey methods. Third Edition. Geneva, Switzerland: World Health Organization; 2004. http://citeseerx.ist.psu.edu/viewdoc/download?doi=10.1.1.378.4797&rep=rep1&type=pdf. Accessed 26 Jan 2019.

[17] Tolonen H (2013). EHES manual. Part B. Field work procedures. Helsinki.

[18] Morisky DE, Green LW, Levine DM (1986). Concurrent and predictive validity of a self-reported measure of medication adherence. Med Care.

[19] World Health Organization. Body mass index – BMI. http://www.euro.who.int/en/health-topics/disease-prevention/nutrition/a-healthy-lifestyle/body-mass-index-bmi. Accessed 26 Jan 2019.

[20] World Health Organization: Obesity and overweight. Fact sheet, updated February 2018. https://www.who.int/news-room/fact-sheets/detail/obesity-and-overweight.

[21] Culig J, Leppée M (2014). From Morisky to hill-bone; self-reports scales for measuring adherence to medication. Coll Antropol.

[22] Lam WY, Fresco P (2015). Medication adherence measures: an overview. Biomed Res Int.

[23] Ben AJ, Neumann CR, Mengue SS (2012). The brief medication questionnaire and Morisky-Green test to evaluate medication adherence. Rev Saude Publica.

[24] Fuller E, Mindell J, Prior G (Ed.). Health Survey for England, 2015. London, England: NHS Digital; 2016. https://www.digital.nhs.uk/catalogue/PUB22616. Accessed 26 Jan 2019.

[25] Ikeda N, Sapienza D, Guerrero R, Aekplakorn W, Naghavi M, Mokdad AH (2014). Control of hypertension with medication: a comparative analysis of national surveys in 20 countries. Bull World Health Organ.

[26] Nulu S (2017). Neglected chronic disease: the WHO framework on non-communicable diseases and implications for the global poor. Glob Public Health.

[27] Ordúñez García P, Pérez Flores E, Hospedales J (2010). Más allá del ámbito clínico en el cuidado de la hipertensión arterial. Rev Panam Salud Publica.

[28] Atun R, Jaffar S, Nishtar S, Knaul FM, Barreto ML, Nyirenda M (2013). Improving responsiveness of health systems to non-communicable diseases. Lancet.

[29] Cooper RS, Ordunez P, Iraola Ferrer MD, Munoz JL, Espinosa-Brito A (2006). Cardiovascular disease and associated risk factors in Cuba: prospects for prevention and control. Am J Public Health.

[30] GreB S, Baan CA, Calnan M, Dedeu T, Groenewegen P, Howson H (2009). Co-ordination and management of chronic conditions in Europe: the role of primary care-position paper of the European forum for primary care. Qual Prim Care.

[31] Guanais F, Macinko J (2009). Primary care and avoidable hospitalizations: evidence from Brazil. J Ambul Care Manage.

[32] Brenes-Camacho G, Rosero-Bixby L (2008). Metabolic control in a nationally representative diabetic elderly sample in Costa Rica: patients at community health centers vs. patients at other health care settings. BMC Int Health Hum Rights.

[33] Cooper RS, Kennelly JF, Ordunez-Garcia P (2006). Health in Cuba. Int J Epidemiol.

[34] World Health Organization. World health statistics 2014. Geneva, Switzerland: WHO Press; 2014. http://apps.who.int/iris/bitstream/10665/112738/1/9789240692671_eng.pdf. Accessed 26 Jan 2019.

[35] Ibrahim MM, Damasceno A (2012). Hypertension in developing countries. Lancet.

[36] Chobanian AV, Bakris GL, Black HR, Cushman WC, Green LA, Izzo JL (2003). The seventh report of the joint National Committee on prevention, detection, evaluation, and treatment of high blood pressure: the JNC 7 report. JAMA.

[37] James PA, Oparil S, Carter BL, Cushman WC, Dennison-Himmelfarb C, Handler J (2014). 2014 evidence-based guideline for the management of high blood pressure in adults: report from the panel members appointed to the eighth joint National Committee (JNC 8). JAMA.

[38] Ettehad D, Emdin CA, Kiran A, Anderson SG, Callender T, Emberson J (2016). Blood pressure lowering for prevention of cardiovascular disease and death: a systematic review and meta-analysis. Lancet.

[39] Hedayati SS, Elsayed EF, Reilly RF. Non-pharmacological aspects of blood pressure management: what are the data? Kidney Int. 2011; 79(10):1061–70. 10.1038/ki.2011.46 PMC3226743 21389976

[40] Oza R, Garcellano M (2015). Nonpharmacologic management of hypertension: what works?. Am Fam Physician.

[41] Melia-Pérez Dania, Castaneda-Abascal Ileana Elena, Pulles-Cuervo Jorge Carlos. Caracterización de pacientes hipertensos no dispensarizados que acuden a un servicio de urgencias. Revista Cubana de Salud Pública (online) 2009; 35(4):128–138. http://scielo.sld.cu/pdf/rcsp/v35n4/spu12409.pdf. Accessed 26 Jan 2019.

[42] Razzaque A, Nahar L, Mustafa AHMG, Karar ZA, Mohammad SI, Yunus M (2011). Sociodemographic differentials of selected noncommunicable diseases risk factors among adults in Matlab, Bangladesh: findings from a WHO STEPS survey. Asia Pac J Public Health.

[43] Taveira LF, Pierin AM (2007). Can the socioeconomic level influence the characteristics of a group of hypertensive patients?. Rev Lat Am Enfermagen.

[44] Morejón Rodríguez W, Achiong Estupiñán F, García Delgado E, Rodríguez López JA, Cárdenas Mederos M. Prevalencia de Hipertensión Arterial y factores asociados. Municipio Matanzas 2009-2010. Rev Méd Electrón (Internet) 2013; 35(5):450–460. http://www.revmedicaelectronica.sld.cu/index.php/rme/article/view/1017/html. Accessed 26 Jan 2019.

[45] Vargas CM, Ingram DD, Gillum RF (2000). Incidence of hypertension and educational attainment: the NHANES I epidemiologic followup study. First National Health and nutrition examination survey. Am J Epidemiol.

[46] Kautzky-Willer A, Dorner T, Jensby A, Rieder A (2012). Women show a closer association between educational level and hypertension or diabetes mellitus than males: a secondary analysis from the Austrian HIS. BMC Public Health.

[47] Lopez-Jaramillo P, Sanchez RA, Diaz M, Cobos L, Bryce A, Parra-Carrillo JZ (2014). Latin American consensus on hypertension in patients with diabetes type 2 and metabolic syndrome. Clin Investig Arterioscler.

[48] Mendis S, Al Bashir I, Dissanayake L, Varghese C, Fadhil I, Marhe E, et al. Gaps in capacity in primary care in low-resource settings for implementation of essential noncommunicable disease interventions. Int J Hypertens 2012; 2012:584041. 10.1155/2012/584041 PMC3517842 23251789

